# Radar Sensing for Intelligent Vehicles in Urban Environments

**DOI:** 10.3390/s150614661

**Published:** 2015-06-19

**Authors:** Giulio Reina, David Johnson, James Underwood

**Affiliations:** 1 Department of Engineering for Innovation, University of Salento, via Arnesano, 73100 Lecce, Italy; 2 Australian Centre for Field Robotics, University of Sydney, Rose Street Building (J04), 2006 Sydney, Australia; E-Mails: d.johnson@acfr.usyd.edu.au (D.J.); j.underwood@acfr.usyd.edu.au (J.U.)

**Keywords:** robotic intelligent vehicles, navigation systems, radar sensing, perception in urban environment, unmanned ground vehicles

## Abstract

Radar overcomes the shortcomings of laser, stereovision, and sonar because it can operate successfully in dusty, foggy, blizzard-blinding, and poorly lit scenarios. This paper presents a novel method for ground and obstacle segmentation based on radar sensing. The algorithm operates directly in the sensor frame, without the need for a separate synchronised navigation source, calibration parameters describing the location of the radar in the vehicle frame, or the geometric restrictions made in the previous main method in the field. Experimental results are presented in various urban scenarios to validate this approach, showing its potential applicability for advanced driving assistance systems and autonomous vehicle operations.

## 1. Introduction

In the past few years, robotic vehicles have been increasingly employed for highly challenging applications including mining, agriculture, search and rescue, planetary exploration, and urban driving. In these applications, the surrounding environment is dynamic and often unknown (*i.e.*, *a priori* information is not available). Under these conditions, in order to enable safe autonomous operations, imaging sensors are required. These sensors can provide obstacle avoidance, task-specific target detection and generation of terrain maps for navigation. Lidar is a common sensing device employed in unmanned robotic vehicles to generate high-resolution maps of the environment. However, due to the near-infrared wavelength they operate at, these sensors are adversely effected in poor weather conditions where airborne obscurants may be present [1]. Similarly, vision sensors are strongly affected by day/night cycles and/or by the presence of environmental factors. Sonar can be a valid alternative for sensing in adverse visibility conditions but it has other issues including noise, atmospheric attenuation, and cross-reflections. Millimetre-wave (MMW) radar overcomes the shortcomings of laser, vision and sonar. The term millimetre-wave in the radar context, refers to electromagnetic radiation with a wave-length ranging between 1 cm and 1 mm. Therefore, MMW radar can operate successfully in the presence of airborne obscurants or low lighting conditions [2]. In addition, radar can provide information of distributed and multiple targets that appear in a single observation. For these reasons, radar perception is increasingly being employed in ground vehicle applications [3].

This paper addresses the general issue of ground detection that consists of identifying and extracting data pertaining to the ground, using a radar sensor, tested in an urban environment. A novel algorithm called Radar-Centric Ground Detection (RCGD) is presented, based on a theoretical model of the radar ground echo, which is used to classify a given sensor reading as either ground or non-ground. Since the RCGD method works directly in the reference frame of the sensor, it does not require a separate synchronised navigation system, nor any calibration with respect to the vehicle reference frame. As will be shown, it also relaxes the geometric assumptions adopted by existing methods in the literature by the same authors for radar-based ground detection.

The ability of detecting and distinguishing ground from non-ground can be studied in different environments. In this research, the main field of interest is the urban environment. Its choice is not related to any application in particular, but arises from the extended variety of cases and problems to be dealt with, as will be discussed further. In general, roads and drivable surfaces are considered to be examples of ground, whereas non-ground is anything different from this: buildings, pedestrians, footpaths, cars, trees, and obstacles in general.

**Figure 1:**
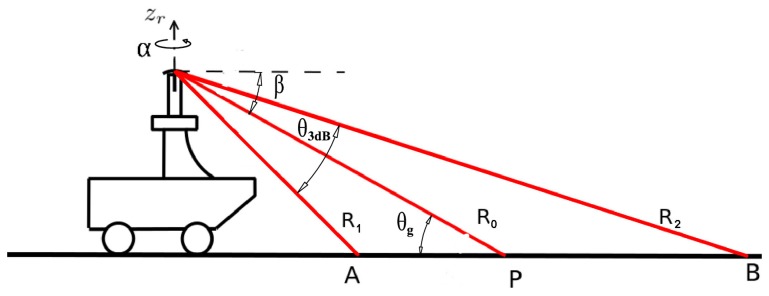
A vehicle equipped with a millimetre-wave (MMW) radar to perform terrain analysis.

**Figure 2:**
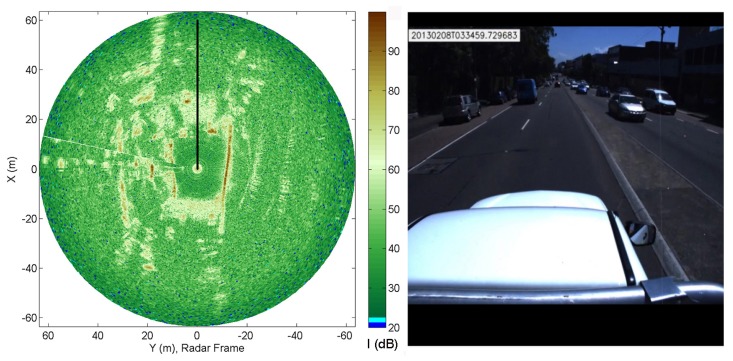
Example of radar image (**Left**). Note that the forward direction of the vehicle is marked by the black line. Front view of the vehicle (**Right**), as acquired by a co-located camera.

The sensing configuration used in this research is shown in Figure 1, where a MMW radar is mounted on the top of the vehicle to survey the surrounding environment. The sensor can sweep around its vertical axis producing a general overview of the environment in a constant time interval (about 0.77 s). Each revolution produces a scan, which is a polar radar image, or plan position indicator (PPI)-scope. An example is shown in Figure 2, where the radar output and the corresponding scene (acquired by an approximately co-located camera) are shown. The forward direction of the vehicle is marked by the black line. Amplitude values above the noise level suggest the presence of objects with significant reflectivity. Amplitude close to or below the noise level generally corresponds to the absence of objects. The radar image provides a clear distinction between road and obstacles of the scene: for instance the spot characterised by high intensity values in the top-left of Figure 2a is the blue van in front of the vehicle in Figure 2b. At a lower range, the white car coming towards the vehicle and the brown car on the red bus lane are also seen respectively to the right and left of the black heading line. The long narrow stripe in the right of Figure 2a is the wall on the right side of the vehicle. Ground areas are visible in front of and behind the vehicle and are characterised by being wide regions with medium intensity values. Clearly, ground appearance in the polar image depends on the relative position between vehicle and road. The further we are from the condition no-roll, no-pitch, flat, horizontal ground, the more difficult to detect the ground, since the difference between obstacles and ground for each beam is less recognisable.

The remainder of the paper is organized as follows. A survey of the literature in the field is provided in Section 2, pointing out the novel aspects of the proposed approach. Section 3 recalls the basic principles of radar sensing and details the adopted ground echo model. The ground classifier is discussed in Section 4, and experimental results obtained with an experimental test bed operating in urban environment are presented in Section 5. Conclusions are drawn in Section 6.

## 2. Related Works

Ground segmentation is the ability of distinguishing ground from other objects that can exist in the scene. In many cases, ground is an unwanted element [4] and many techniques have been provided in order to eliminate its effects [5]. On the other hand, ground detection is a key requirement for autonomous navigation [6]. Recent sensor developments (Velodyne, Riegl, Ibeo, *etc.*) have led to increased perceptual ability. For instance, laser is still the main sensor used to survey the three-dimensional (3D) shape of terrain [7] and its comparatively high resolution makes it the preferred choice for obstacle avoidance [8]. Computer monocular- and stereo-vision is used if lighting conditions are appropriate; for vehicular applications this typically restricts operations to daylight hours [9–11]. Relatively limited research has been devoted to investigate the use of MMW radar for ground vehicle applications. In [12], a radar has been developed for the measurements in ore-passes and to survey the internal structures of dust and vapour filled cavities. Radar capability to perceive the environment has been demonstrated in challenging conditions [13], including a polar environment (Antarctica) [14]. Radar sensing has also been employed for autonomous guidance of cargo-handling vehicles [15] and occupancy mapping [16]. In [17], the combination of radar with monocular vision is proposed within a statistical self-supervised approach. In the automotive field, various perception methods have been proposed that mostly use radar technology for collision avoidance and obstacle detection [18–20].

Several works from the literature dealt with the problem of detecting ground, but few of them employed radar as the main sensor. One of them, previously proposed by the authors [21] and called Radar Ground Segmentation (RGS), represents the starting-point of this research. The RGS algorithm consists of two steps:

- Prediction of the expected location of the ground within the sensor data by using a 6-DOF localisation system. This provides the starting point for the ground search within the radar data. This step is called background extraction.
- Labelling of each radar observation as ground, obstacles or uncertain, according to the goodness of fit to the ground-echo model. This is the ground segmentation step.

It should be noted that the background extraction is a necessary but not sufficient condition for a given observation to be labelled properly. This means that if the first part of the algorithm fails, the second part will inevitably fail too. It can happen if one of the assumptions made in the RGS algorithm is not verified. As shown later, transporting the algorithm from a rural to an urban environment causes some of the original assumptions to be degraded.

The main contribution of this work is a novel method, namely the RCGD algorithm that is able to detect the ground surfaces within radar scans of the environment, while relaxing the geometric assumptions made by the previous algorithm. Specific differences between this research and the previous RGS method are highlighted throughout the paper and in particular in Sections 3.2 and 3.3.

## 3. Principles of Radar Sensing

In this section the basic mathematics that describe radar sensing is recalled. The theoretical model of the radar ground echo that is used in this research is, then, developed in detail.

### 3.1. Radar Fundamentals

Keeping in mind the scheme of Figure 1, under the assumption of a pencil (narrow) beam and uniformly illuminated antenna, it is possible to describe the beam-pattern by a cone of aperture *θ*_3_*_d__B_*, which is the half-power beam-width. The cone intersects the ground with a footprint delimited by the proximal and the distal border denoted as a and B, respectively *R*_1_ and *R*_2_ are the proximal and distal range, whereas *R*_0_ is the bore-sight range in which the peak of the radiation is concentrated. The angle of intersection between the bore-sight and ground is denoted by the grazing angle *θ_g_*. Radar uses electromagnetic energy that is transmitted to and reflected from the reflecting object. The radar equation expresses this relationship. The received energy is a small fraction of the transmitted energy. Specifically, the received energy by the radar can be calculated as

(1) $$P_{r}=\left(\frac{P_{t}G}{4\pi R^{2}}\right)\cdot \left(\frac{\sigma_{t}}{4\pi R^{2}}\right)\cdot \left(\frac{G\lambda^{2}}{4\pi }\right)$$

where *P_t_* is the transmitted power, *G* the antenna gain, *λ* the wave length, *σ_t_* the radar cross section, and *R* the range. The first grouped term in Equation (1) represents the power density (watts per square meters) that the radar transmitter produces at the target. This power density is intercepted by the target with radar cross section *σ_t_*, which has units of area (square meters). Thus the product of the first two terms represents the reflected power density at the radar receiver (again, watts per square meters). The receiver antenna, then, collects this power density with an effective area described by the third term. It is possible to combine all the constant terms and obtain:

(2) $$P_{r}=k\frac{G^{2}\sigma_{t}}{R^{4}}$$

Usually, the reflectivity *σ*_0_ in place of *σ_t_* is preferred

(3) $$\sigma_{0}=\frac{\sigma_{t}}{A_{c}}$$

*A_c_* being the clutter area or footprint. It can be shown that *A_c_* depends on the range *R* and on the secant of the grazing angle *θ_g_*. Thus combining all constant terms in *k′*, Equation (2) can be rewritten as

(4) $$P_{r}=k^{\prime }\frac{G^{2}\sigma_{0}\sec\theta_{g}}{R^{3}}$$

This equation can be converted to *dB*:

(5) $$P_{rdB}=K+20\log\left(G\right)+10\log\left(\sigma_{0}\sec\left(\theta_{g}\right)\right)-30\log\left(R\right)$$

### 3.2. Ground Model

Equation (5) shows that the ground echo depends only on *θ_g_*, *R*, and *G*. In turn, the gain *G* can be expressed as a function of the bore-sight *R*_0_ and grazing angle. Specifically, *G* is maximum when the target is located along the antenna's bore-sight, and it reduces with angle off bore-sight, as defined by the antenna's radiation pattern. If a Gaussian antenna pattern is adopted, an elevation angle *θ_el_*, measured from the radar bore-sight, can be defined as

(6) $$\theta_{el}=\arcsin\left(\frac{R_{0}\sin\theta_{g}}{R}\right)-\theta_{g}$$

The antenna gain can then be approximated by [22] as

(7) $$G=e^{-2.776{\left(\frac{\theta_{el}}{\theta_{3dB}}\right)}^{2}}$$

*θ*_3_*_dB_* being the 3dB beamwidth (*θ*_3_*_dB_* = 3 deg for the radar used in this research). This will allow to write Equation (5) as

(8) $$P_{rdB}=f\left(R,R_{0},\theta_{g}\right)$$

Equation (5) is the ground-echo model used in this research. The main difference with the model previously proposed in [21] is that the grazing angle *θ_g_* is not treated as a constant but it can change as a function of the terrain inclination.

Equation (5) is completely defined once *K* is known. *K* can be obtained, for example, by evaluating *P_rdB_* at *R*_0_, as proved in the Appendix. Equation (5) defines the ground echo in terms of power return. However, the location or range spread of the ground echo can also be predicted, based on geometric considerations (please refer again to Figure 1). The proximal and distal borders can be expressed as a function of *θ_g_* and bore-sight *R*_0_

(9) $$\begin{array}{c} R_{1}=\frac{R_{0}\sin\theta_{g}}{\sin\left(\theta_{g}+\frac{\theta_{3dB}}{2}\right)}\\ R_{2}=\frac{R_{0}\sin\theta_{g}}{\sin\left(\theta_{g}-\frac{\theta_{3dB}}{2}\right)} \end{array}$$

Equation (5) must be interpreted in this way: given a ground radar observation, *P_rdB_*(*R*_0_) is known. The other terms *G*, *σ*_0_ and *K* depend only on *R*_0_ and the grazing angle. The dependence is complex, therefore the couple (*R*_0_, *θ_g_*) can be found only by investigating all the possible couples and comparing each corresponding RCGD ground-echo model against the radar observation until a satisfactory agreement is reached.

**Figure 3:**
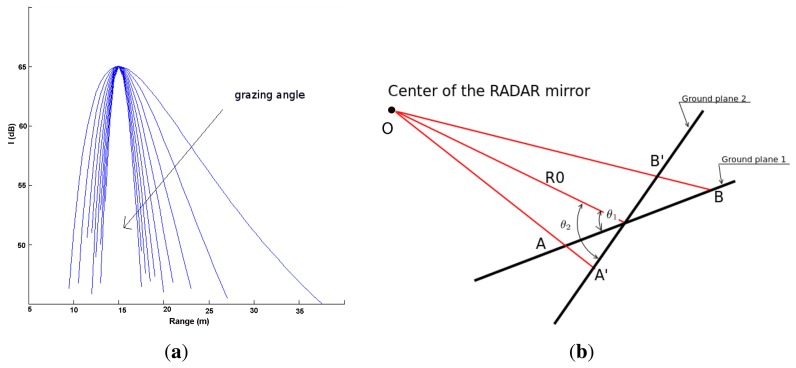
Change in the ground-echo model for increasing grazing angles, as indicated by the arrow in figure (*R*_0_ = 15 *m*, *I_R_*__0__ = 65 *dB*), *θ_g_* = 3−12° (**a**); Change in the footprint and range-spread as a function of *θ_g_* while *R*_0_ is kept constant (**b**).

In order to evaluate the validity of the RCGD ground-echo model, Figure 3a shows the change of the model as a function of the grazing angle, whereas *R*_0_ is maintained constant. As the grazing angle increases, the RCGD ground-echo model becomes narrower and the range spread decreases. This is in line with Figure 3b; the first configuration denoted as ground plane 1 is characterised by the grazing angle *θ*_1_, whereas the second is denoted as ground plane 2 and it is characterised by the grazing angle *θ*_2_: *θ*_2_ > *θ*_1_, whereas *R*_0_ is the same for both configurations. It is apparent that the range spread (*R*_2_ − *R*_1_) for the first configuration (*OB* − *OA*) is higher than the range spread of the second configuration (*OB′* − *OA′*). Indeed, increasing the grazing angle leads to a higher *R*_1_ (*OA′* > *OA*) and a lower *R*_2_ (*OB′* < *OB*).

### 3.3. Comparison with Existing Literature

**Figure 4:**
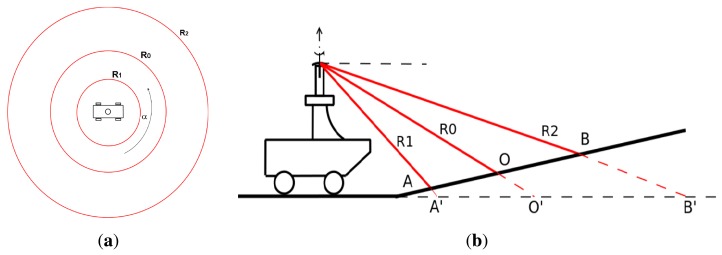
Radar beam model for flat, horizontal ground, up view (**a**); Uphill ground in view of the radar (**b**).

Previous RGS method in this field showed that, knowing the pose of the vehicle and the characteristics of the radar beam, it is theoretically possible to locate the ground footprint. For example, for the radar configuration used in this work and under the assumptions of quasi-horizontal ground, the area illuminated by the radar on the terrain is shown in Figure 4a. Clearly, the effect of roll and pitch will be to deform the three annuli (stretching them along roll and pitch axes resulting in three ellipses). The main assumptions underlying the previously proposed RGS method can be summarized as follows

- The radar position relative to the vehicle reference frame is known by initial calibration and fixed during travel.
- The pose (six degree of freedom position and orientation) estimation of the vehicle with respect to the ground is available from an onboard navigation system.
- There are negligible time synchronisation errors between the navigation solution and the radar data.
- Approximately horizontal ground in view of the radar.
- The ground plane is coincident with the plane of vehicular motion.

These assumptions pose some limitation to the RGS system, leading to the following main reasons of failure

- The assumption of quasi-horizontal ground is too restrictive. For example, in urban environment, several uphill and downhill roads exist.
- An onboard navigation system is not always available.
- Faster vehicle dynamics (e.g., higher speeds and sharper bumps) magnify errors due to time synchronisation.
- The plane of vehicular motion is not always coincident with the plane of the area to be detected.

The first reason of failure is the presence of a climb or a descent or, more generally, when the ground plane is different from the plane of the vehicular motion. Figure 4b shows that the background location prediction of the RGS is A′O′B′, which differs from the real plane AOB.

There is another aspect to be discussed. The background extraction step of the RGS method requires the knowledge of the calibration parameters in order to take into account the relative position between the radar and the vehicle. If they are not exact and some errors are present, there will be a double systematic error in the results: the first is in the background extraction itself and the second is in the projection of the results in the world reference frame. Later, it will be shown that the RCGD algorithm does not require the calibration parameters for the ground detection (even if they are still necessary to project the results in the world reference frame).

## 4. The Radar-Centric Ground Detection Algorithm

**Figure 5:**
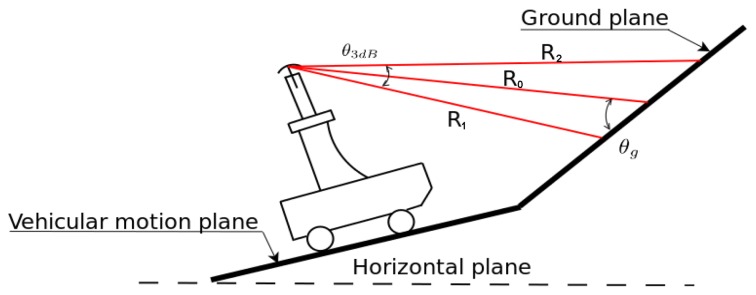
Radar-based ground detection in a general environment where motion plane and area under investigation do not coincide.

A novel ground detection algorithm, referred to as the RCGD algorithm, is presented that operates directly in the sensor frame of the radar, without the need for a separate synchronised navigation source or any geometric restriction of the area investigated. Let us refer to the general case where the plane of vehicular motion is not coincident with the ground plane in view of the radar. In addition, the two planes can be freely tilted in the space and no assumption is made about their location. These observations lead to the representation in Figure 5. Therefore, the main idea is that the ground-echo depends only on the information described by the sensor frame and the relative position between the bore-sight and the ground plane, defined by the grazing angle. This is suggested by Equation (5), which is theoretically independent of the navigation solution.

In order to define a classification scheme to label radar data pertaining to the ground, a given radar observation (*i.e.*, the single radar reading obtained for a given scan angle or azimuth angle) is compared with the ground model. The underlying hypothesis is that a good fit between the theoretical model and experimental data suggests high confidence of ground. Conversely, a poor fit would indicate low likelihood of ground due, for example, to an obstacle.

### 4.1. Fitting the Ground Echo Model

In Section 3.2, it was proved that the ground-echo model can be expressed as a function of the bore-sight *R*_0_, and the grazing angle *θ_g_*. Moreover, once these parameters are defined, the ground echo model is unique. In this section, it is explained how to use the ground echo model to detect ground areas. The problem is to find the location or range spread (*R*_1_, *R*_2_) and the couple (*R*_0_, *θ_g_*) that defines the RCGD ground-echo model, which best fits the sensory data. In the RCGD algorithm, no assumption is made, therefore the search for *R*_0_ can be performed theoretically by an exhaustive iteration over the whole plausible range of values. However, the search is limited in practice to the interval 8-22 m. The upper bound can be explained when considering that the assumption of near-field region is violated beyond this limit and Equation (5) loses its validity. The lower bound is chosen based on the maximum road slope expected in urban environments. The search for *θ_g_* is based on geometric considerations related to the inclination of the vehicle and of the road. In principle, an interval of 0−90° can be chosen, but this means a high computational time. In this approach, the variation range of the grazing angle was limited to 2–15° with an increment of 0.5°, to match the limits of the gradients in our urban test environment. This presents an optimisation problem, which could be solved with a variety of standard optimisation procedures. In this work, we chose to limit the search bounds and employ exhaustive search.

For a given radar observation, the objective is to find the model that best fits the experimental data. In order to investigate the set of all possible pairs (*R*_0_,*θ_g_*), two loops are performed by varying *R*_0_ and *θ_g_*, respectively, in their interest range. Each iteration defines a candidate RCGD ground model according to Equation (5), which can be fitted against this radar reading. The corresponding goodness of fit can be evaluated by the squared error (SE). The candidate (*R̅*_0_,*θ̄_g_*) resulting in the lowest SE is retained as the RCGD ground model for the given radar observation

(10) $$SE\left(j\right)=\sum_{p=1}^{W}{\left(I_{j}\left(p\right)-P_{rdB}\left(p\right)\right)}^{2}$$

where:

- *SE*(*j*) is the lowest squared error evaluated at the *j* – *th* radar observation
- *W* is the the number of points in the window of interest (defined by Equation (9))
- *I_j_*(*p*) is the *p* – *th* intensity value within the window of interest of the *j* – *th* radar observation
- *P_rdB_*(*p*) is the RCGD ground echo model defined by the best couple (*R̅*_0_,*θ̄_g_*) evaluated at the *p*–*th* point within the window of interest

**Figure 6:**
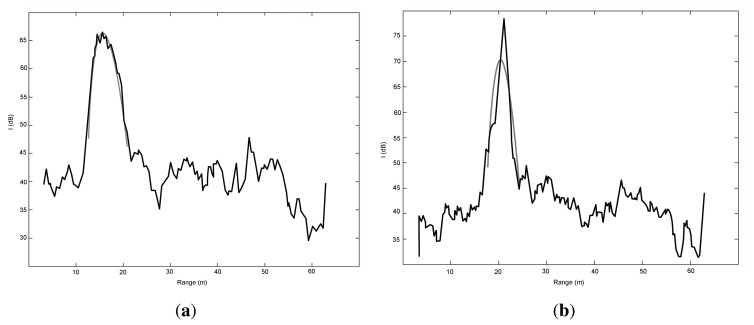
Example of good fit (SE = 94.0 *dB*^2^) (**a**), and poor fit (SE = 913.1 *dB*^2^) (**b**). Black: radar observation. Grey: Radar-Centric Ground Detection (RCGD) ground echo model.

The idea is that a good fit (*i.e.*, low SE) between the RCGD model and experimental data indicates high likelihood of ground, whereas a poor fit (*i.e.*, high SE) indicates low confidence in ground. Two sample results are plotted in Figure 6. Specifically, in Figure 6a, the model matches the experimental data very well, thus attesting to the presence of ground. Conversely, Figure 6b shows an example where the SE is high; in this case a low confidence in ground echo is associated with the given observation. In practice, a threshold *SE_th_* is determined by inspection, and the observation *j* is labeled as ground if *SE*(*j*) exceeds *SE_th_*.

**Figure 7:**
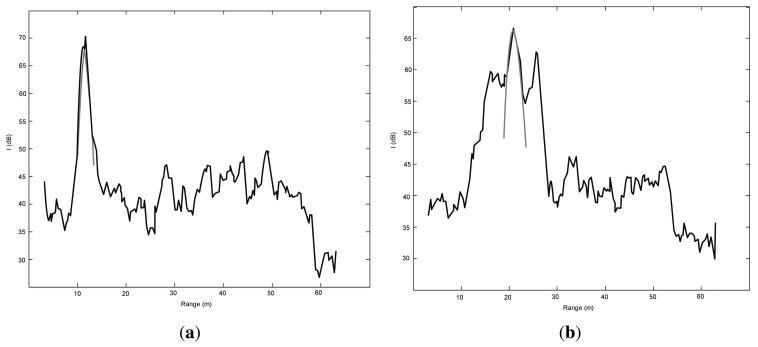
Examples of false positives: an obstacle would be erroneously labeled as ground considering only the SE (**a**); an obstacle in the vicinity of ground would be flagged as ground (**b**). Black: radar observation. Grey: RCGD ground echo model.

However, relying on the squared error only, may lead to many false positives, *i.e.*, obstacles that are erroneously labelled as ground. An example is shown in Figure 7a, where a ground return would be seemingly detected based on the value of SE, which results lower than the threshold and comparable to a ground observation, when there is actually an obstacle. Nevertheless, it can be observed that data fitting gets worse in the proximity of *R*_0_, due to the difference in the peak value between the radar observation and the ground echo model. This suggests a possible solution to this issue by defining an absolute change in the maximum intensity value between the radar observation *I_max_* and the model $P_{rdB}^{\max}$, $\Delta P=\left|\left(I_{\max}-P_{rdB}^{\max}\right)\right|$. Therefore, non-ground will be flagged by the system when Δ*P* exceeds an experimentally defined threshold Δ*P_th_*.

It should also be noted that obstacles are generally characterised by higher reflection values than ground radar observations. Thus, a check can be performed by considering $P_{rdB}^{\max}$, which is the peak of the RCGD ground-echo model obtained from a given radar observation. This radar observation will be labelled as non-ground if $P_{rdB}^{\max}$ exceeds an experimental threshold *P_th_*.

Finally, Figure 7b shows a second type of false positive, due to the simultaneous presence of ground and obstacle. The obstacle “attracts” the algorithm, which labels erroneously the observation as ground with an *R*_0_ equal to the peak of the obstacle signal. An additional check is effective in this occurrence. The range spread Δ*R* = (*R*_2_ − *R*_1_) of the RCGD ground echo model results narrower than that of the experimental data. Therefore, an observation is denoted as non-ground if its range spread is lower than a threshold, Δ*R_th_*, defined by inspection as will be explained in Section 4.2.

In summary, Table 1 collects the classification rules chosen for the algorithm. Those rules represent our physical understanding of the problem and they are not unique. Indeed, other rules may be conceived and implemented to improve the performance of the system.

### 4.2. Setting the Thresholds for the Classification Rules

**Figure 8:**
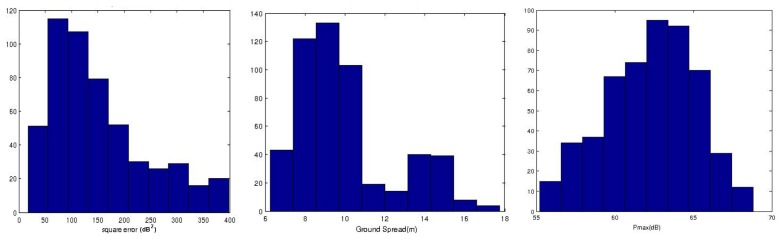
Histogram of the ground model features in the training set: squared error (Left), range spread (**Center**), maximum intensity (**Right**).

*N* observations were manually labelled by verifying against the corresponding frames provided by the camera. They were further divided into a training set of size *M*, which was used for setting the thresholds of the classification rules, and an independent group of size *Q* for evaluation, as will be explained later in Section 5. The squared error, range-spread Δ*R*, and $P_{rdB}^{\max}$ values of the ground-labeled returns in the training set were stored and the results are illustrated in Figure 8. These plots show the variability of these parameters. They also demonstrate that no ground observation was detected with a squared error higher than 400 *dB*^2^, a range-spread less than 6 m and a $P_{rdB}^{\max}$ higher than 68 *dB*. These values are the chosen thresholds for the classification rules of Table 1. The Δ*P* threshold was manually tuned by observing the resulting classified obstacles and a similar process of manual verification using the camera images as a reference. The threshold chosen was 3 *dB*.

## 5. Results

**Figure 9:**
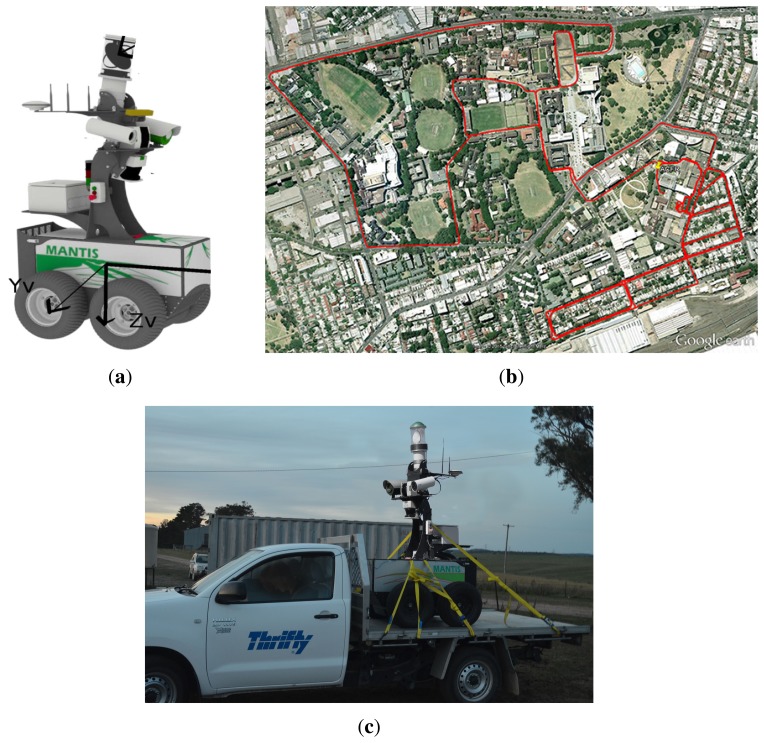
The experimental platform Mantis (**a**); urban path followed during the experiment (**b**); performed by fastening Mantis on a manned utility vehicle (**c**).

In this section, experimental results are presented to validate the RCGD algorithm. The system was tested in the field using Mantis (Figure 9a), a mobile platform fastened on a manned utility vehicle (Figure 9c). On the top of Mantis is mounted the HSS radar employed in this research. The sensor, custom built at the Australian Centre for Field Robotics (ACFR), is a 95 GHz frequency-modulated continuous wave (FMCW) radar that reports the amplitude of echoes at range between 1 and 60 m. The wavelength is λ = 3.16 mm and the 3-dB beam-width is 3° in azimuth. The antenna scans across the angular range of 360°. The main sensor properties are collected in Table 2. The robot is also equipped with other sensors, including one 2D Sick laser range-scanner, a colour camera, and a GPS/INS unit that provides pose estimation of the vehicle during the experiments.

The test field is a path around the Redfern Campus (The University of Sydney, NSW, AU), and it is visible in Figure 9b. Along the path, the vehicle encountered various obstacles (cars, buildings, people), slopes and climbs. During the experiment, radar images were collected and stored for processing offline.

**Figure 10:**
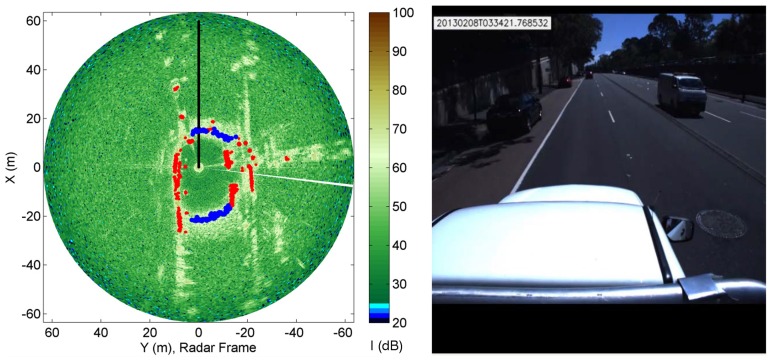
Classification results obtained from the RCGD method overlaid over the radar image (**Left**) in a typical urban environment (**Right**). Blue dot: Radar-labelled ground. Red dot: Radar-labeled obstacle.

A typical result obtained from the RCGD approach is shown in Figure 10, referring to time *t* = 22.31 min, when the vehicle drove on a double-lane road in regular traffic. Blue dots are the given observations labelled as ground, whereas red dots denote radar-labelled obstacles. The road in front of and behind the vehicle, as well as the car on the right and the car and the wall on the left are properly detected.

**Figure 11:**
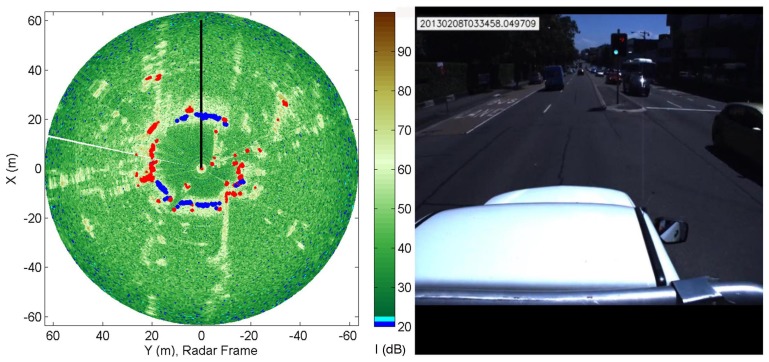
Output of the RCGD algorithm overlaid over the radar image (**Left**) in a cluttered urban environment (**Right**). Blue dot: Radar-labelled ground.

Figure 11 shows another result obtained in a cluttered environment with many cars simultaneously present in the scene. The algorithm succeeds also to detect ground areas between the several obstacles in the scene. In this scene blue dots are the given observation labelled as ground. Everything else not denoted by blue spots is an obstacle.

**Figure 12:**
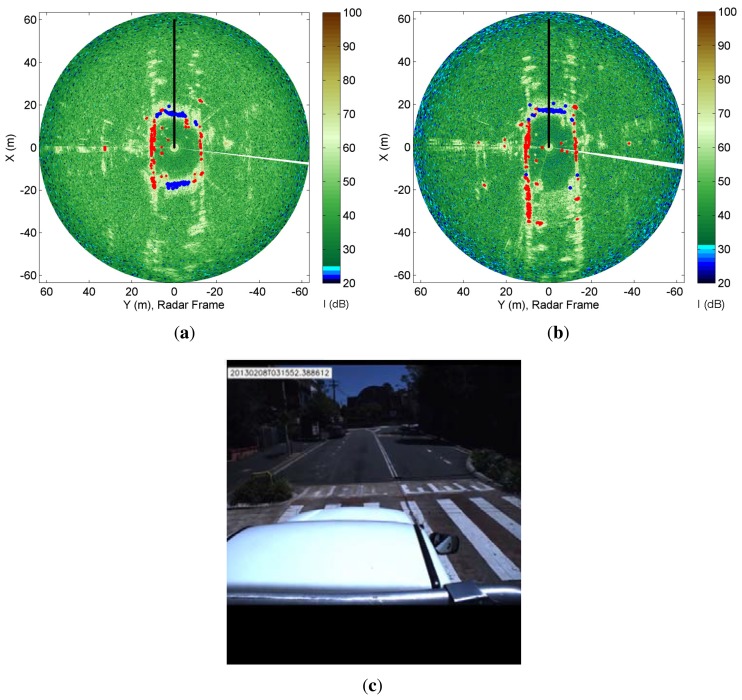
Sequence of the radar images superimposed with the RCGD results (**a**) and (**b**); Results obtained from the RCGD method after the traverse of a hump (**c**).

Figure 12 refers to two consecutive frames during the negotiation of a hump. The RCGD algorithm is not affected by time-synchronisation errors, as previously explained, and the ground areas are adequately detected even in the presence of pitch oscillations. It should be noted that in Figure 12b the rear ground section is not detected, due to the low grazing angle.

Overall, the data set used for the RCGD testing consisted in 87 labelled frames. Note that only radar observations that fall within the camera field of view and can therefore be hand-labeled by visual inspection were considered (*i.e.*, about 8000 observations). A quantitative evaluation of the system performance was obtained by measuring detection rates, precision, recall and F1-score [23]. The results are collected in Table 3. It resulted in a true positive rate (the fraction of ground observations which were correctly classified as ground) of 86.0% and a false positive rate (the fraction of non-ground observations, which were erroneously classified as ground by the system) of 3.3%. The overall accuracy, *i.e.*, the fraction of correct detections with respect to the total number of classifications, was of 90.1%.

As a final remark, it should be recalled that, since the RCGD approach operates in the radar reference frame, it does not require any navigation system. In contrast, this was a requirement for the previous RGS method. This also made impossible a direct comparison of the two methods, due to the relatively low accuracy and large local rifts in the navigation solution caused by multi-path and satellite occlusions in the urban “canyons”.

## 6. Conclusions

This paper addressed the issue of radar-based perception of autonomous vehicles in urban environments. The use of the radar for the perception of the surrounding environment is a recent research area which is strictly correlated to the available technologies. Specifically, the radar used in this thesis is a MMW radar and this choice is manly due to the demand of good resolution required to the sensor. Moreover, although the algorithm addresses specifically urban environments, this research provides a general overview of the field and can inspire other works for different environments. A novel method to perform ground segmentation was proposed using a radar mounted on a ground vehicle. It is based on the development of a physical model of the ground echo that is compared against a given radar observation to assess the membership confidence to the general class of ground or non-ground. The RCGD method was validated in the field via field experiments showing good performance metrics. This technique can be successfully applied to enhance perception for autonomous vehicles in urban scenarios or more generally for ground-based MMW radar terrain sensing applications.

Future research will focus on the possible extension of the RCGD approach to other similar sensor modalities, the use of statistical learning approaches to automatically build the ground model, and obstacle classification using, for example, intensity and geometric features (e.g., wall-angle).

## Figures and Tables

**Table 1:** Classification rules of the RCGD algorithm. Refer to Section 4.2 for more details on the thresholds.

**Class**	**Parameters of the Regression Model**			
	*SE_th_* = 400 *dB*^2^, Δ*P_th_* = 3 *dB*			
	*P_th_* = 68 *dB*, Δ*R_th_* = 6 *m*			
	*SE*	Δ*P*	*P*	Δ*R*
Ground (all conditions must be verified)	< *SE_th_*	< Δ*P_th_*	<*P_th_*	> Δ*R_th_*
Non-ground (one condition must be verified)	≥ *SE_th_*	≥ Δ *P_th_*	≥ *P_th_*	≤ *R_th_*

**Table 2:** HSS radar technical properties

	**Model**	**Max. Range**	**Raw Range Resolution**	**Horizontal FOV**	**Instantaneous FOV**	**Angle Scan Rate**	**Angular Resolution**
Radar	ACFR custom-built	60 m	0.15 m	360°	3.0 × 3.0°	≃1.75 rps	0.77°

**Table 3:** Classification results obtained from the RCGD method for a subset of salient images.

	**RCGD**
True positive rate	86.0%
False positive rate	3.3%
True negative rate	96.7%
Precision	97.1%
Accuracy	90.1%
F1-score	90.7%

## Appendix

If the intensity value (in dB) at *R*_0_ is known (*P_rdB_*(*R*_0_)), it is possible, from Equation (5), to write:

(A1) $$P_{rdB}\left(R_{0}\right)=10\log\left(\sigma_{0}\left(\theta_{g}\right)\sec\left(\theta_{g}\right)\right)-30\log\left(R_{0}\right)+K$$

where 20*log*(*G*(*R*_0_)) = 0 because *G*(*R*_0_) = 1, and *σ*_0_(*θ_g_*) is the value of *σ*_0_ for *θ_g_*. The only unknown in the previous equation is *K*, then:

(A2) $$K=P_{rdB}\left(R_{0}\right)-10\log\left(\sigma_{0}\left(\theta_{g}\right)\sec\left(\theta_{g}\right)\right)+30\log\left(R_{0}\right)$$

It should be noted that, according to the frequency of the radar used in this research (*i.e.*, 94 Hz), the reflectivity can be also assumed as a function of the grazing angle *θ_g_*

(A3) $$\sigma_{0}=10\log\left(a{\left(\theta_{g}+c\right)}^{b}\right)$$

Please refer to [24] for more information on the value of the parameters involved.
